# Transcriptome based identification and validation of heat stress transcription factors in wheat progenitor species *Aegilops speltoides*

**DOI:** 10.1038/s41598-021-01596-6

**Published:** 2021-11-11

**Authors:** Sushmita Seni, Satinder Kaur, Palvi Malik, Inderjit Singh Yadav, Parul Sirohi, Harsh Chauhan, Amandeep Kaur, Parveen Chhuneja

**Affiliations:** 1 School of Agricultural Biotechnology, Punjab Agricultural University, Ludhiana, Punjab 141004 India; 2 Department of Biosciences and Bioengineering, Indian Institute of Technology Roorkee, Roorkee, Uttarakhand 247667 India

**Keywords:** Biotechnology, Computational biology and bioinformatics, Molecular biology, Plant sciences

## Abstract

Wheat, one of the major cereal crops worldwide, get adversely affected by rising global temperature. We have identified the diploid B genome progenitor of wheat, *Aegilops speltoides* (SS), as a potential donor for heat stress tolerance. Therefore, the present work was planned to study the total transcriptome profile of heat stress-tolerant *Ae. speltoides* accession pau3809 (AS3809) and compare with that of tetraploid and hexaploid wheat cultivars PDW274 and PBW725, respectively. The comparative transcriptome was utilized to identify and validate heat stress transcription factors (HSFs), the key genes involved in imparting heat stress tolerance. Transcriptome analysis led to the identification of a total of 74 K, 68 K, and 76 K genes in AS3809, PDW274, and PBW725, respectively. There was a high uniformity of GO profiles under the biological, molecular, and cellular functions across the three wheat transcriptomes, suggesting the conservation of gene function. Twelve HSFs having the highest FPKM value were identified in the AS3809 transcriptome data, while six of these HSFs namely *HSFA3*, *HSFA5*, *HSFA9*, *HSFB2a, HSFB2b,* and *HSFC1b,* were validated with qRT PCR. These six HSFs were identified as an important component of thermotolerance in AS3809 as evident from their comparative higher expression under heat stress.

## Introduction

Wheat is the world's second most important source of nutritional energy and nutrients, after rice. Despite being the world's second-largest wheat producer, yields have been decreasing for several years, notably in India’s central regions^1^. Climate change on a global scale, particularly the current anomalous temperature regimes, poses a serious threat to the agricultural environment. Given future climatic uncertainty and resource limits, providing food and nutritional security for an expanding population will be a huge challenge^2^.

With an increase in ambient temperature since the turn of the century, global climate models project a rise in mean ambient temperatures ranging from 1.8 to 5.8 °C by the end of the century^3^. Heat stress has the most adverse effect on productivity as wheat flowers at cool temperatures between 0 and 5 °C. Although heat stress affects wheat growth to varying degrees at different phenological stages, its effects are more prominent on reproductive development (also called terminal heat stress) than on vegetative growth as an increase in temperature during grain filling leads to a reduction in grain filling duration, which directly impacts grain number and yield^4^. Previous studies^5^ have estimated grain filling duration and grain yield to decrease by 5% and 3–4%, respectively, with a per degree increase in temperature between 18 and 22 °C during grain filling. Based on this calculation, losses up to 50% in yield potential on exposure to 32–38 °C during the critical grain formation period have been estimated^6^.

Wheat belongs to the family Poaceae, tribe *Triticeae* and is placed in the genus *Triticum*. *Triticum* comprises diverse types of cultivated and wild species of wheat. The genome size of bread wheat (*Triticum aestivum* L.), a cultivated allohexaploid species is about 16,000 Mb and contains three different genomes (AABBDD), while durum is a cultivated tetraploid species (AABB) of approximately 12,000 Mb. Origin of wheat passed through a complex pathway involving the crossing of three different genomes followed by their localization in a single nucleus. These genomes though different from each other but are related in their evolutionary pathways. The B genome of the bread wheat and durum wheat has been derived from a closely related diploid species of wild wheat *Aegilops speltoides* (SS), but this probably happened only once or a few times and involved only a few progenitors. Consequently, the potential genetic diversity of *Ae. speltoides* have not been represented well in bread and durum wheat germplasm^7^.

The importance of wild species, particularly, *Aegilops* species in breeding programs is documented by various studies where they have a role in improvement for tolerance to drought stress^8^, heat stress^9,10^ and salinity stress^11^, resistance to several pests and diseases such as rusts^12–14^ and also have contribution towards traits as complex as yield^7^. Therefore, besides being a progenitor of bread wheat, *Ae. speltoides* has established itself as a valuable genetic resource for wheat improvement. Also, the ability of this wild progenitor to adapt to different climatic zones including drought/heat environments, different disease hot spots, and nutrient-poor areas has enhanced its value as a breeding resource.

Heat stress transcription factors (HSFs) regulate core aspects of the heat stress response^15^ by turning on almost all "heat shock genes" (HSGs) thus, protecting against heat stress. Several heat-inducible genes known as HSGs are up-regulated in response to heat stress and encode heat shock proteins (HSPs) which protect intracellular proteins from denaturation^16^. The presence of conserved heat shock elements (HSEs) in the promoter region of HSGs initiates transcription in response to heat. Plant HSFs are a complex gene family having an important role in the modulation of transcription during heat stress^17^. Based on details of their oligomerization domains, plant HSFs have been distinguished into three classes, class A, class B, and class C HSFs^15^. Only A-type HSFs show transactivation property due to the presence of a C-terminal AHA type activation domain, while B-type HSFs have been proposed as either co-activators or repressors. Multiple copies of HSFs have been reported in plants: 21 in *Arabidopsis*, 24 in tomato, 25 in pepper, and 56 in wheat^18–21^.

Previous studies have highlighted the importance of HSFs in wheat under various abiotic stress conditions, particularly heat stress^21–23^. A seed preferential HSF; *TaHSFA2d* now annotated as *TaHSFA6b* was cloned for the first time in wheat^23^ and its overexpression in *Arabidopsis* was reported to increase thermotolerance. The induction of *HSFA2*, *HSFA6* in response to heat stress in wheat^21^, and high expression of *TaHSFC2a* gene during grain filling and transient induction in the leaves by heat stress have also been reported^24^. Overexpression of *TaHSFC2a-B* in transgenic wheat is reported to cause upregulation of thermo-protectant genes-*TaHSP70d* and *TaGalSyn*.

The diploid *Ae. speltoides* (BB)*,* known to be closely related to the species which contributed the B genome to tetraploid and hexaploid wheat. At Punjab Agricultural University (PAU), India, we have a collection of about 160 accessions of *Ae. speltoides* and preliminary investigations led to the identification of this species as a source of heat stress tolerance as evident by its stay-green trait, normal pollen fertility, and normal seed setting at temperatures as high as 39 °C^6^. We also developed a number of lines carrying introgressions from different accessions of *Ae. speltoides* to the durum and hexaploid wheat background under our wheat-wide hybridization program. The present study was designed with an aim to understand the variations in the HSF genes offered by the heat-tolerant *Ae. speltoides* accession pau 3809 (AS3809) over the two cultivated but heat susceptible cultivars, PBW725 (hexaploid, bread wheat) and PDW274 (tetraploid, durum wheat). This analysis provides fundamental insights into HSFs and their variants available in AS3809 which would be utilized in the wheat improvement programs for heat stress tolerance.

## Results

### Transcriptomic data filtering and de novo assembly

A comprehensive overview of the transcriptome in three different wheat ploidies yielded 16, 21, and 18 million paired-end reads for AS3809, PBW725, and PDW274, respectively. A summary of statistics of de novo assemblies is given in Table 1. After removing adapter sequences, ambiguous and low-quality reads, the high-quality clean reads (Phred score ≥ 30) led to the assembly of AS3809 with 113,157 trinity transcripts corresponding to 74,946 genes. 121,466 and 109,827 trinity transcripts from PBW725 and PDW274, respectively corresponding to 76,404 and 68,873 genes. N50 values of 1409, 1398, and 1004 bp for AS3809, PBW725, and PDW274 respectively, and average GC content ranging from 47.57 to 48.22%, indicated the good quality of all the assemblies, suitable for further annotation (Table 1).

**Table 1:** Summary of paired-end sequencing data (2 × 100 bp) and de novo transcriptome assembly statistics for *Ae. speltoides* acc. pau3809, *T. aestivum* cv. PBW725 and *T. durum* cv. PDW274.

Feature	*Aegilops speltoides* acc. pau3809	*Triticum aestivum* cv. PBW725	*Triticum durum* cv. PDW274
Total number of raw paired-end reads	16,991,059	21,158,755	18,808,576
Paired-end reads obtained after filtering	13,397,450 (78.85%)	16,480,554 (77.89%)	13,824,303 (73.50%)
Number of trinity transcripts	113,157	121,466	109,827
Number of trinity ‘genes’	74,946	76,404	68,873
GC content (%)	47.57	48.11	48.22
Contig N50 (bp)	1409	1398	1004
Average contig length (bp)	809.01	850.16	796.23

### Assessment of transcriptome assembly completeness

BUSCO analysis revealed that the majority of the Liliopsida core genes were successfully recovered in the AS3809 assembly (Table 2). Specifically, of the 3278 single-copy orthologs searched, 74.3% of core genes were completely recovered in AS3809 as compared to 65.4% and 56.7% in PBW725 and PDW274, respectively. Around 12.1%, 14%, and 16% of genes in AS3809, PBW725, and PDW274, respectively were partially recovered in fragmented form. Only 13.6% of genes were missing from AS3809 assembly as compared to 16.1% and 19.4% missing rate for hexaploid and tetraploid wheat. Given the high quality of the dataset, recovery for both ‘complete-single copy’ and ‘complete-duplicated’, BUSCOs was considerably higher for AS3809 assembly as compared to PBW725 and PDW274. Therefore, the AS3809 transcriptome was used as the standard for extracting transcripts related to heat stress tolerance.

**Table 2:** Summary of the complete, duplicated, fragmented and missing orthologs inferred from BUSCO searched against the 3278 single-copy orthologs for Liliopsida.

BUSCO statistics	*Aegilops speltoides acc. pau* 3809 (AS3809)	*Triticum aestivum* (PBW725)	*Triticum durum* (PDW274)
Complete BUSCOs (C)	2436 (74.3%)	2144 (65.4%)	1859 (56.7%)
Complete and single-copy BUSCOs (S)	1534 (46.8%)	1373 (41.9%)	1272 (38.8%)
Complete and duplicated BUSCOs (D)	901 (27.5%)	754 (23.0%)	679 (20.7%)
Fragmented BUSCOs (F)	396 (12.1%)	459 (14.0%)	524 (16.0%)
Missing BUSCOs (M)	445 (13.6%)	528 (16.1%)	636 (19.4%)

### Annotation and Gene Ontology analysis

**Figure 1:**
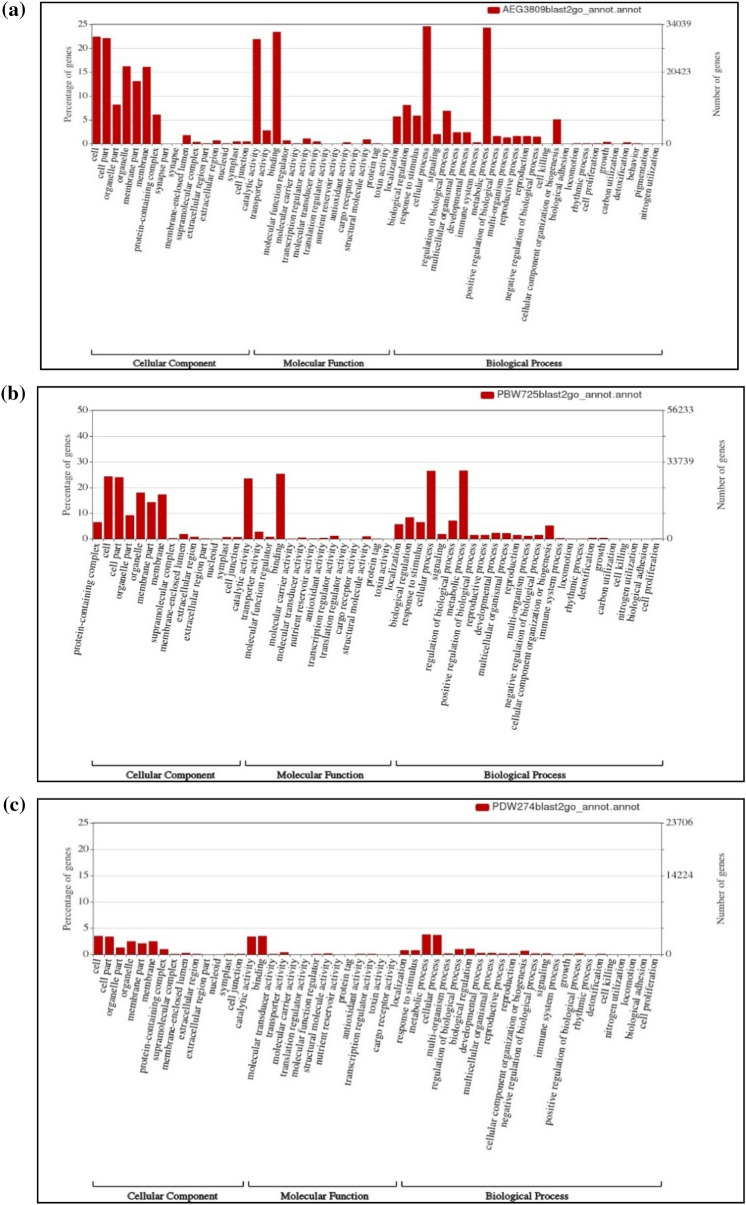
Proportion of gene ontology annotations made using WEGO for transcripts of (**a**) *Aegilops speltoides* acc. pau3809, (**b**) *T. aestivum* cv. PBW725 and (**c**) *T. durum* cv. PDW274 representing a cellular component, molecular function, and biological process.

Blast2GO annotation based on the BLASTX results led to functional characterization of 55,838, 50,077, and 49,872 transcripts in AS3809, PBW725, and PDW274, respectively. All the assemblies exhibited a diverse range of GO, suggesting that biological process, molecular function, and cellular component were all well represented (Fig. 1). These three main categories were further divided into 53 GO functional subcategories uniformly across the three transcriptomes. In all the assemblies, genes encoding cellular and metabolic processes along with the response to stimuli were significantly represented in the biological process category while under the molecular function category, the percentage of genes coding for binding and catalytic activities was higher. Within the cellular components, proteins related to cell and cellular parts were expressing more, followed by the cellular membrane and organelles.

Functional terms were also assigned to the trinity transcripts from each wheat assembly using the MapMan Mercator. MapMan employs a basic hierarchical tree structure of terms called "bins" to represent biological contexts and concepts. MapMan annotation resulted in the classification of the transcripts into different bins, each bin representing a different group as photosynthesis, carbohydrate metabolism, glycolysis, gluconeogenesis, oxidative phosphorylation, TCA, cell wall synthesis, lipid and amino acid metabolism, biotic and abiotic stress response, etc. The abiotic stress bin revealed that the majority of the active transcripts were from redox state, respiratory, cell wall, MAPK, defense genes, PR proteins, heat shock proteins, heat shock transcription factors, auxin, hormone signaling, etc. in all three assemblies. The PfamScan categorized transcripts into domains, families, and repeats with all maximum in AS3809 and minimum in PDW274. The annotation statistics obtained for the three assemblies are shown in Table 3.

**Table 3:** Summary of transcripts with significant BLAST2GO alignments and annotation using annotation tools of MapMan Mercator and PfamScan.

Annotation	*Ae. speltoides* acc. pau3809	*T. aestivum* cv. PBW725	*T. durum* cv. PDW274
BLAST2GO	55,838	50,077	49,872
**MapMan mercator annotations**			
Total mapped transcripts	103,683	118,104	99,565
Transcript specific for abiotic stress	772	823	689
**Pfam scan based on HMM**			
Number of domains	26,386	24,992	19,321
Number of families	19,748	18,820	16,242
Number of repeats	5127	4239	3403

### Identification of orthologous genes

Ortholog transcript detection, based on the OrthoMCL program, demonstrated considerable overlap in transcripts sequences across all three assemblies. Over 39% of the transcripts were identified as putative orthologs between AS3809 and PBW725 assemblies while PDW274 and AS3809 assemblies shared around 37% of the transcripts as putative orthologs. The annotation results from BLAST2GO, MapMan, and PfamScan of the ortholog transcripts were similar suggesting the authenticity of identified ortholog.

### Identification of putative heat shock transcription factor (HSF) genes

**Figure 2:**
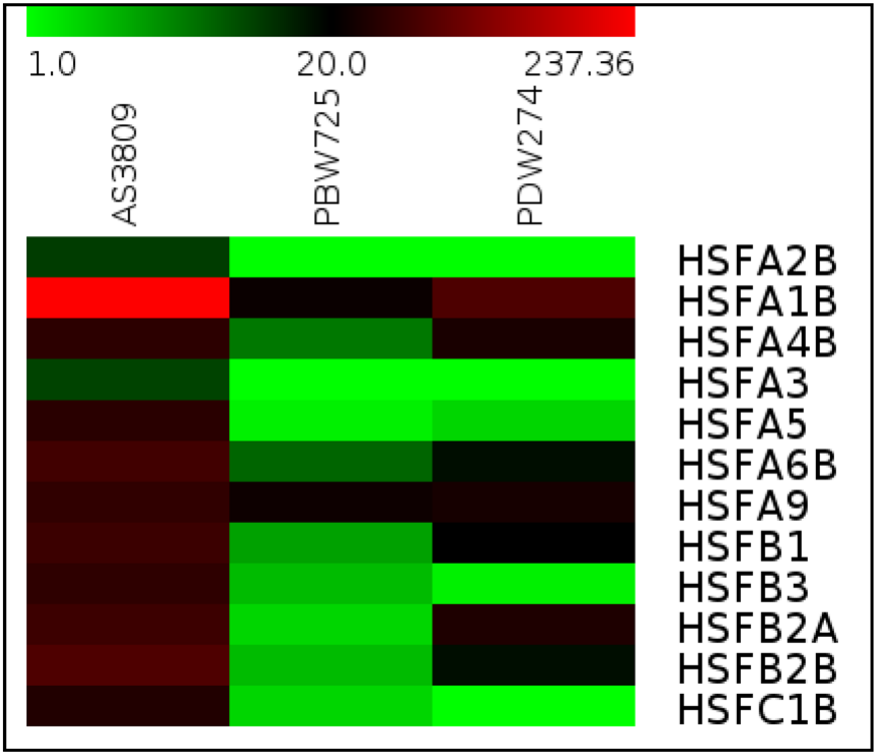
Heat map representing the different classes of HSF transcripts having a similar function in AS3809, PBW725, and PDW274 (red: maximum expression, black: median, green: minimum expression).

The three different classes of HSF i.e., A, B, and C were annotated/identified from the transcripts. In total, 37 HSF transcripts were retrieved from AS3809 assembly along with their orthologs from PDW274 and PBW725. Of these, 12 HSFs viz*., HSFA1b, HSFA2b*, *HSFA4b*, *HSFA3*, *HSFA5*, *HSFA6b*, *HSFA9*, *HSFB1*, *HSFB3*, *HSFB2a*, *HSFB2b* and *HSFC1b* showing maximum FPKM values were chosen for further analysis. All these 12 HSFs had higher expression in AS3809, followed by PDW274 and the least expression value in PBW725 (Fig. 2). The selected HSF genes have high sequence similarity (95–98%) among three wheat species and possessed HSF DNA binding domain as evident from Pfam analysis. Gene Ontology from Blast2GO and PfamScan annotation of these HSF transcripts are given in Table 4.

**Table 4:** List of Trinity IDs of 12 HSFs selected from *Aegilops speltoides* acc. pau3809 transcriptome assembly with maximum FPKM value along with their GO annotation.

S. no	AS3809 transcript ID	HSF subtype	Length (bp)	FPKM value	GO annotation	PfamScan annotation
1	TRINITY_DN7915_c1_g2_i3	*HSFA2b*	688	15.51	AHB61248.1heat shock factor HsfA2b	HSF_DNA-bind (Domain)
2	TRINITY_DN1997_c0_g4_i2	*HSFA1b*	2257	237.36	AHZ44763.1heat shock factor A1b	HSF_DNA-bind (Domain)
3	TRINITY_DN7915_c1_g1_i1	*HSFA4b*	2357	58.73	XP_020165982.1heat stress transcription factor A-4b	HSF_DNA-bind (Domain)
4	TRINITY_DN49987_c0_g1_i1	*HSFA3*	660	55	XP_020146599.1heat stress transcription factor A-3-like	HSF_DNA-bind (Domain)
5	TRINITY_DN16884_c0_g1_i1	*HSFA5*	1562	56	XP_020200706.1heat stress transcription factor A-5	HSF_DNA-bind (Domain)
6	TRINITY_DN4362_c0_g1_i10	*HSFA6b*	2640	77	AHZ44769.1heat shock factor A6b	HSF_DNA-bind (Domain)
7	TRINITY_DN3035_c0_g1_i2	*HSFA9*	1521	62	XP_020148069.1heat stress transcription factor A-9-like	HSF_DNA-bind (Domain)
8	TRINITY_DN4144_c0_g1_i2	*HSFB1*	1280	71.4	XP_020177659.1heat stress transcription factor B-1-like	HSF_DNA-bind (Domain)
9	TRINITY_DN4144_c0_g1_i5	*HSFB3*	2471	60.23	BAJ96942.1predicted protein	HSF_DNA-bind (Domain)
10	TRINITY_DN4527_c0_g1_i1	*HSFB2a*	1628	72	AJR10106.1heat-responsive transcription factor	HSF_DNA-bind (Domain)
11	TRINITY_DN4527_c0_g2_i1	*HSFB2b*	1800	88	XP_020163127.1heat stress transcription factor B-2b-like	HSF_DNA-bind (Domain)
12	TRINITY_DN32883_c0_g1_i1	*HSFC1b*	786	49	XP_020179067.1heat stress transcription factor C-1b-like	HSF_DNA-bind (Domain)

### In silico expression analysis of HSF genes (tissue-specific and under abiotic stress)

**Figure 3:**
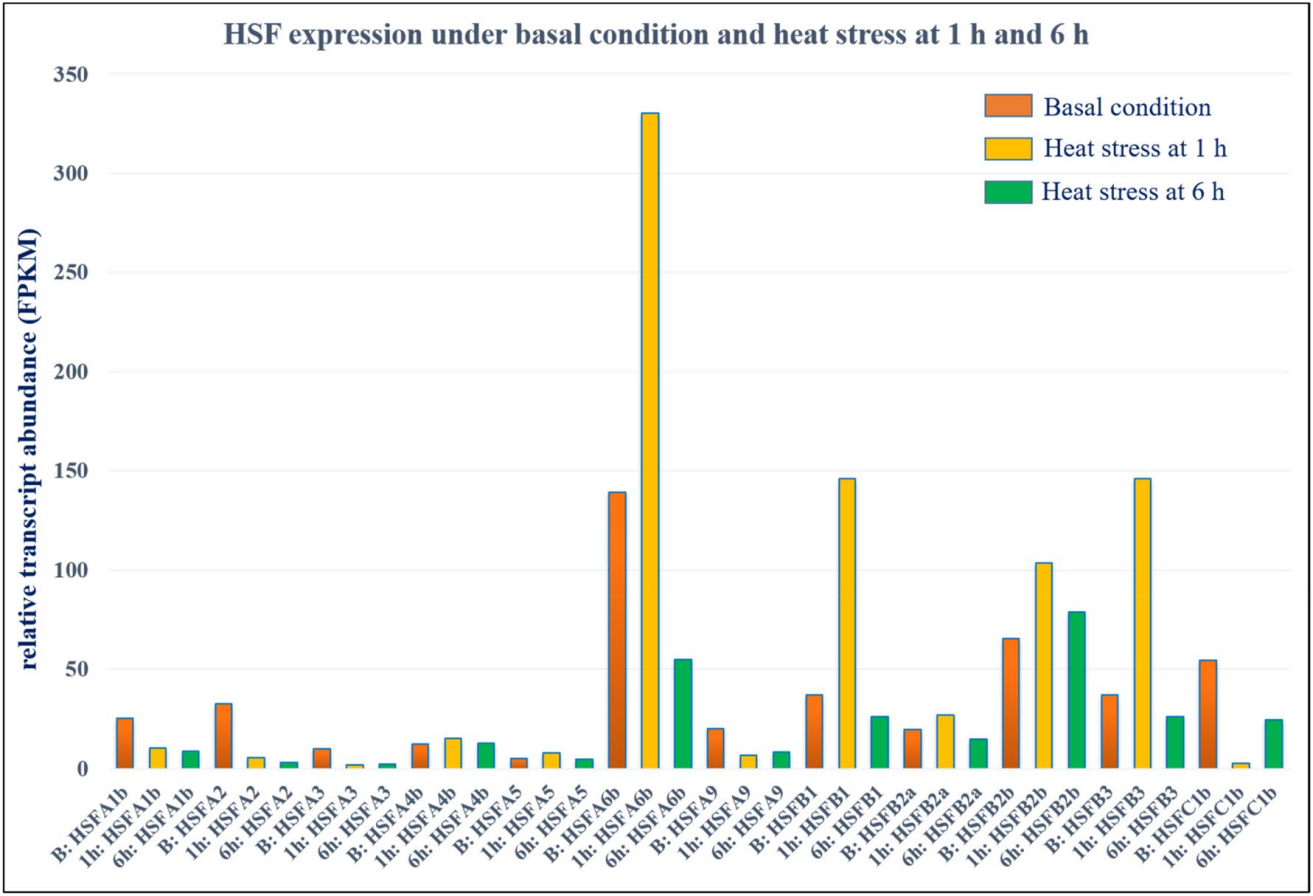
In silico gene expression analysis of 12 HSFs from the transcriptome assemblies in wheat expression database (WheatEXP) represented in the figure as B: basal conditions, 1 h: heat stress at 1 h, and 6 h: heat stress at 6 h.

BLASTn search of selected 12 HSF transcripts with the wheatEXP database suggested expression levels of all the HSF genes to be significantly higher in leaf (Z71) tissues. At the basal level, the expression of *HSFA6b* was found to be the highest, followed by *HSFB2b* and *HSFC1b* (Fig. 3). Similarly, at 1 h of heat stress, the expression of *HSFA6b* was the highest, followed by *HSFB3*, *HSFB1,* and *HSFB2b*, indicating these genes play regulatory functions and be induced in plants within 1 h of heat stress. At 6 h of heat stress, the expression of *HSFB2b* was found the highest amongst all 12 HSFs. But in the expression database, the expression values recorded under prolonged heat stress (6 h) were less than those observed under 1 h of heat stress.

### Protein–protein interaction (PPI) network analysis of the HSF proteins

**Figure 4:**
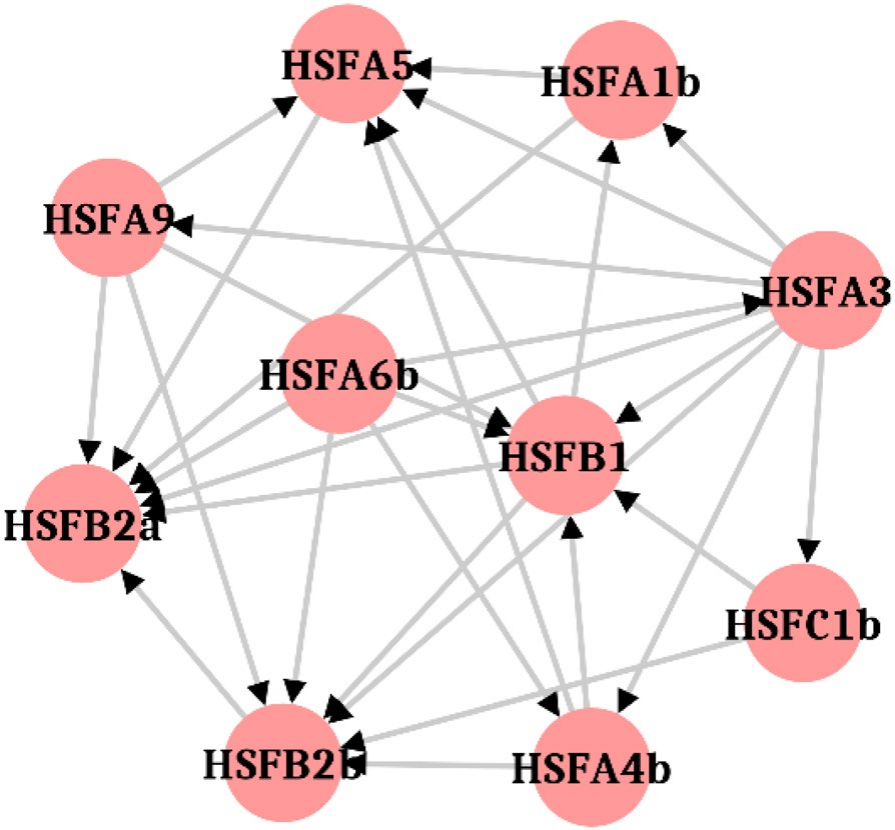
Protein–protein interaction (PPI) network analysis exhibiting interaction of HSF proteins with one another. In the network, the nodes correspond to the proteins and the edges represent the interactions. Arrows represent the interaction between one HSF protein with another by degree value. The source and points of arrows show the sources and targets of HSF protein in the network. More arrows at a node mean more number of HSF proteins are interacting with that particular HSF.

Protein–protein interaction network data was represented in the form of two distinct variables; node, representing a protein, and an edge, representing the interaction between two proteins. A highly connected network of selected HSFs was observed with 10 nodes representing 10 selected HSFs and 30 interactions in form of edges. This PPI network revealed that there is a complex network of interaction between different HSFs as *HSFC1b* and *HSFA9* interact only with *HSFA3* while *HSFA1b* and *HSF4b* has two interactions each. *HSFA5* and *HSFB1*were more interactive with five interactions each. But almost all the HSF proteins interactions end to *HSFB2a* (7 edges) and *HSFB2b* (6 edges). This reflected that *HSFB2a* and *HSFB2b* could be the primary regulators of the heat shock response and their response might have been modulated by other HSFs selected in the present study. *HSFA6b* on other hand seems to be a mediator, interacting with the other proteins to modulate their functions, rather than directly affecting the heat shock response (Fig. 4).

### Phylogenetic relationship among HSF gene family

**Figure 5:**
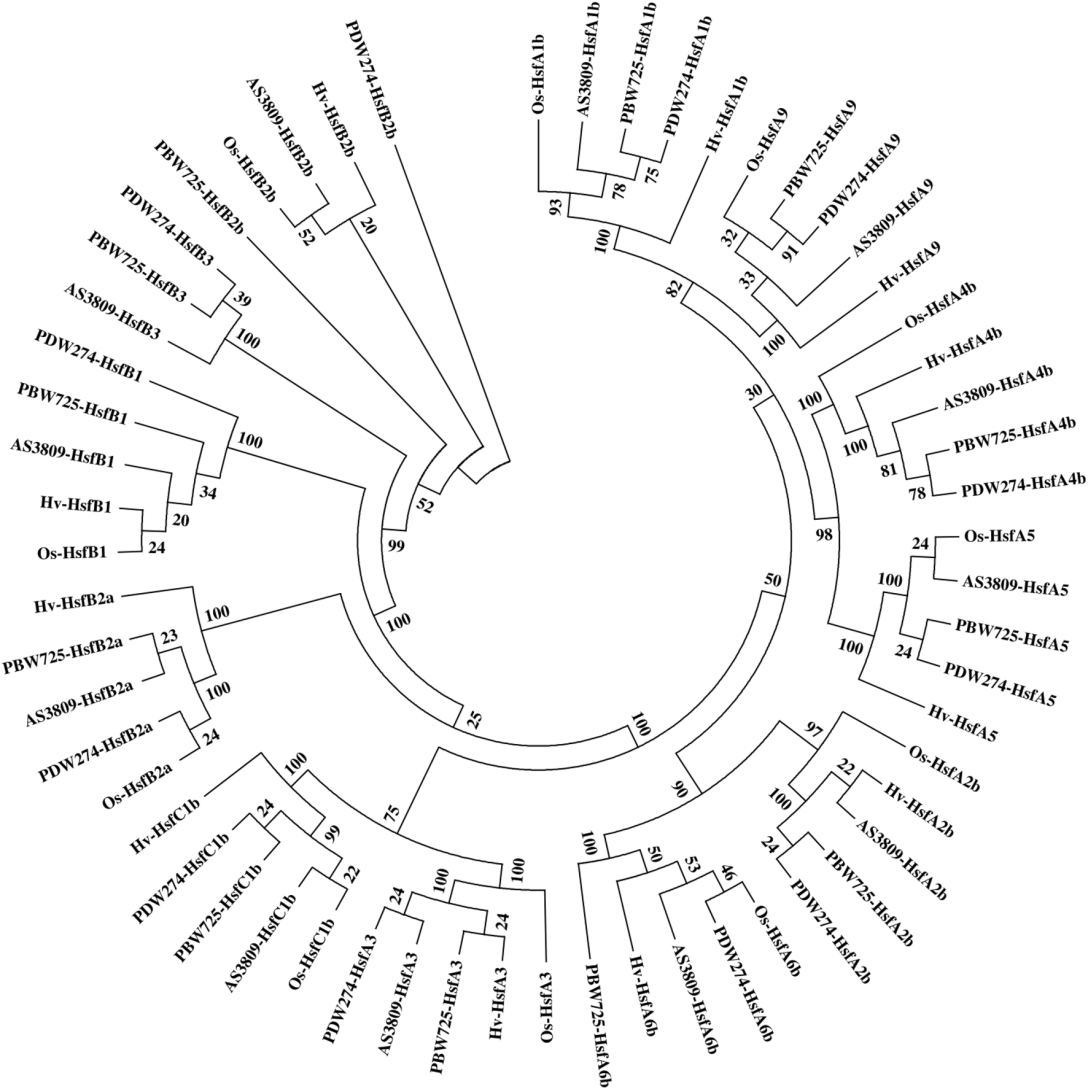
Phylogenetic analysis of HSF gene family of rice, barley, diploid, tetraploid, and hexaploid wheat. Numbers represent bootstrap values.

To find the evolutionary relationship and potential roles of wheat HSF genes based on known functions of barley and rice HSFs^25^, an unrooted neighbor-joining (NJ) phylogenetic tree was constructed using Mega 7.0 (Fig. 5). As expected, 12 wheat HSF proteins identified in the present study were clustered closely with respective HSFs from rice and barley, and clearly separated into two main groups. The first group contained the HSF proteins from the B class and was further clustered into two main sub-groups named Ia (comprising of *HSFB2b*, *HSFB3,* and *HSFB2a*) and Ib (comprising of *HSFB1*). AS3809-*HSFB2b* was found to be close to Os-*HSFB2b* while highly diverged from PBW725-*HSFB2b*. For *HSFB2*a and *HSFB1* proteins, wheat proteins were observed to have close relatedness with each other but were distinct from the barley and rice proteins.

However, the second cluster contained the A and C classes of HSF proteins and was subdivided into two groups, named group IIa and IIb. The IIa cluster contained *HSFC1b* and *HSFA3* proteins and in both these clusters, rice and barley proteins were observed to have less similarity with the wheat proteins. *HSFA3* clustered separately from the rest of the class A present in cluster IIb and showed close relatedness with the *HSFC1b* gene. On the other hand, cluster IIb represented a group of two sub-clusters, having *HSFA5* and *HSFA4b* (ClusterIIb.1), while the second sub-cluster (IIb.2) contained the rest of the four HSF protein categories (*HSFA6b*, *HSFA2b*, *HSFA9,* and *HSFA1b*).

### Validation of the expression of HSF genes

**Figure 6:**
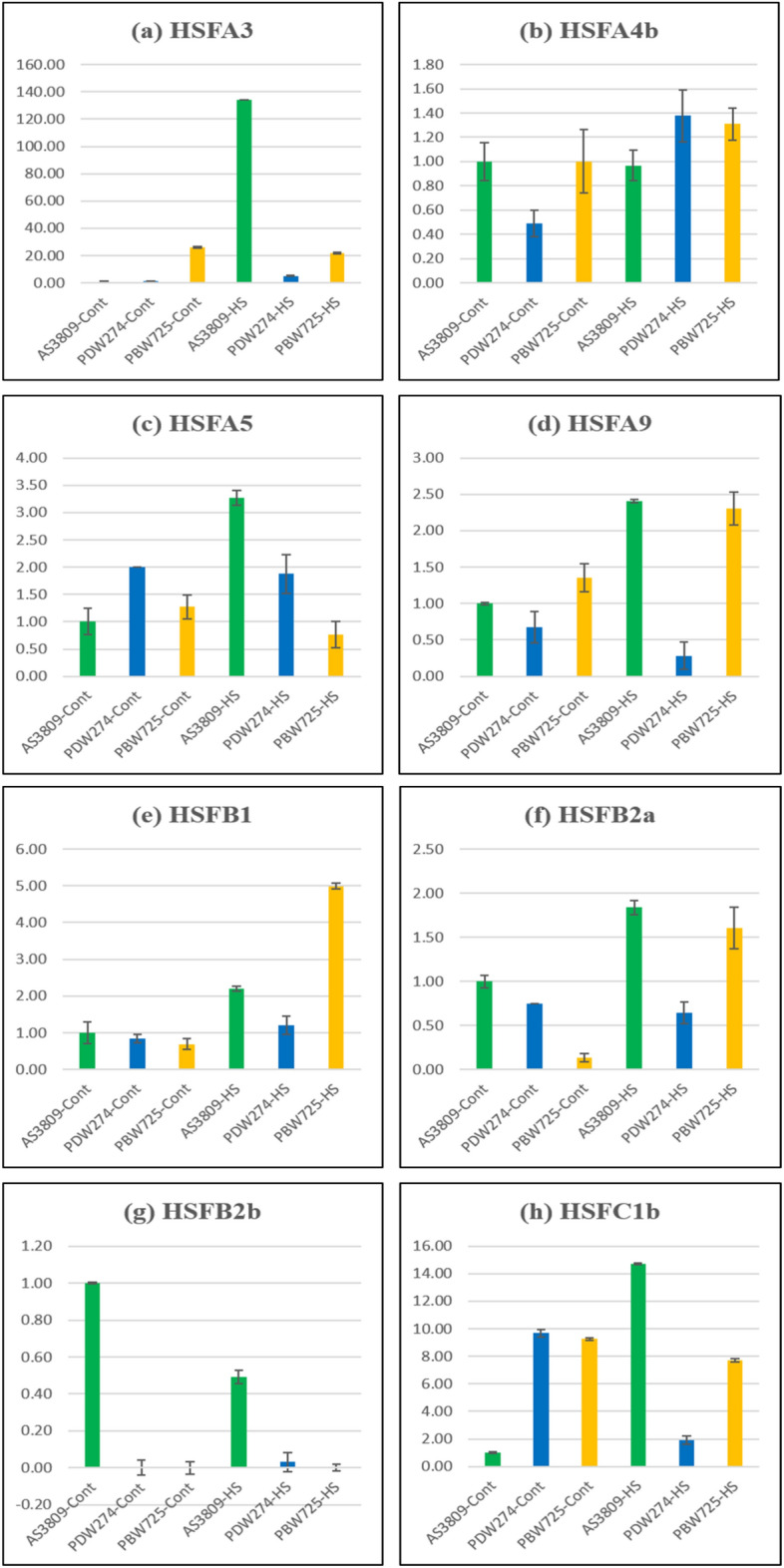
Expression analysis of HSF genes in response to control and high-temperature stress in three species of wheat (AS3809, PBW725, and PDW274). Change in transcript abundance of HSF genes in 10 days old seedlings of wheat species of different ploidy levels subjected to heat stress at 35 °C for 1 h. (**a**) *HSFA3* (**b**) *HSFA4b* (**c**) *HSFA5* (**d**) *HSFA9* (**e**) *HSFB1* (**f**) *HSFB2a* (**g**) *HSFB2b* (**h**) *HSFC1b*. The expression level at control (AS3809) i.e., 0 h of treatment was normalized as 1.0. The results shown are the means ± SE of three independent experiments.

Of the 12 selected HSFs, eight have been validated through qRT-PCR, as four (*HSFA1b*, *HSFA2b*, *HSFA6b,* and *HSFB3*) failed to amplify in two or more selected wheat species (Fig. 6). After 1 h of heat stress on seedlings, *HSFA3*, *HSFA5*, *HSFA9*, *HSFB2a,* and *HSFC1b* exhibited the highest expression in the wild wheat species AS3809 while two HSF genes, *HSFA4b* and *HSFB1* exhibited high expression in cultivated durum PDW274, and hexaploid wheat PBW725 respectively. *HSFB2b* though amplified in control but did not show expression after heat stress.

## Discussion

Heat stress is one of the most serious constraints that limit wheat productivity. The mechanism of heat stress tolerance is a complicated phenomenon governed by numerous genes which cause a variety of physiological and biochemical changes. Wild species always fascinate scientists for having a repertoire of exotic genes/alleles which have almost vanished in cultivated wheat due to domestication and breeding^8,10,12–14^. Various studies have highlighted the importance of *Ae. speltoides*, *Ae. tauschii* and *Ae. geniculata* being a valuable genetic resource for enhancing thermotolerance^9,26,27^. Previous studies^6,28^ reported *Ae. speltoides* to be highly heat-tolerant species, which can be utilized to enhance the thermotolerance of wheat^27^. Therefore, our study was designed to identify the unique transcript from the selected accession AS3809 by comparing it with the cultivated tetraploid and hexaploid wheat and study the expression profiling of selected HSFs among three ploidies.

### Transcriptome data analysis of wild and cultivated wheat

De novo transcriptome assembly was performed for all three transcriptomes using Trinity, to avoid any bias arising due to the non-availability of reference genomes of *Ae. speltoides* and *T. durum*. Regardless of the huge difference in the genome size (4.9–7 GB)^29–31^ and the number of genes (50,000–100,000)^30,32^ among the wild diploid, cultivated tetraploid and hexaploid wheat, the total number of expressed transcripts obtained were 113.1 K in AS3809 which is just marginally less than hexaploid wheat PBW725 having 121.4 K and tetraploid PDW274 with 109.8 K transcripts. The disproportionate trends between genome size and gene expression have been reported in polyploid species owing to the momentous number of repetitive sequences in the non-expressing heterochromatin region of higher ploidies as compared to diploid relatives. Furthermore, loss of some genes/functional alleles during artificial selection and domestication seems to play a direct role in the observed trend^33,34^.

The N50 values obtained for the three transcriptome assemblies were found to be appropriate for downstream analysis^35,36^. BUSCO analysis-based completeness assessment of the three transcriptomes revealed the highest recovery of conserved single-copy orthologs in the diploid wild followed by hexaploid and tetraploid wheat, indicating good coverage and high quality of AS3809, relative to higher ploidies, demonstrating loss of genes owing to the domestication process as well as polyploidy events^37^. Recovery reported for the assemblies was enough for identification of single-copy orthologs^38^ thus the current assemblies of the cultivated as well as wild species of wheat will supplement the published resources for wheat. BLAST2GO and MapMan based comprehensive annotation revealed that GO representations for all three categories of molecular function, biological processes, and cellular component were successfully captured representing GO profiles with the highest proportions of mapped GO terms^39^.

### Identification and validation of HSFs

AS3809 shared 39% and 37% putative orthologs with PBW725 and PDW274 respectively. The transcriptome data have been analyzed for differential expression of putative HSF gene candidates for heat stress tolerance. HSFs play an imperative role in acquired thermotolerance as they are the terminal components of the signal transduction chain mediating the activation of genes responsive to heat stress^16^. The 12 important HSF genes under study had the highest FPKM in wild wheat indicating higher expression in AS3809 as compared to cultivated durum and hexaploid wheat. These HSFs are primarily involved in stimulating the rapid synthesis and accumulation of heat shock proteins (HSPs), which not only act as molecular chaperones shielding proteins from thermal aggregation but are also involved in various aspects of proteins homeostasis, such as protein translocation and degradation^40^.

The selected HSFs from the transcriptome data were validated with *in-silico* expression analysis data from the WheatExp database and with real-time qRT-PCR. Of the 12 important HSF genes with highest expression in AS3809, eight viz., *HSFA3*, *HSFA4b*, *HSFA5*, *HSFA9*, *HSFB1*, *HSFB2a*, *HSFB2b* and *HSFC1b* were validated by qRT-PCR, (Fig. 6). Of these 6 genes *HSFA3*, *HSFA5*, *HSFA9*, *HSFB2a, HSFB2b*, and *HSFC1b* showed comparative highest expression in the wild AS3809 as proven in comparative transcriptome data. But two important HSFs, *HSFA6b* and *HSFB3* having a very good expression in response to heat stress in the WheatExp database, could not be validated by qRT-PCR. In the WheatEXP database, *HSFA6b* had higher expression at basal level and after 1 h of heat stress, *HSFB1* and *HSFB3* after 1 h of heat stress, and *HSFC1b* having higher expression at a basal level only. All HSF genes in the WheatEXP database revealed that the expression values recorded under prolonged heat stress of 6 h were less than those observed under 1 h of heat stress suggesting the HSF gene family be quickly induced within 1 h of heat stress, activating downstream pathways for thermotolerance as compared to 6 h of heat stress, thus allowing a quick and elevated response system of plants to combat heat stress. *HSFC1b* and *HSFB2b* might have a key regulatory role in thermotolerance in AS3809. Higher expression of *HSFC1b* in transcriptome data and qRT-PCR and the WheatExp database results indicating this to be important HSF in AS3809. *HSFB2b* could be another important candidate though it expressed only under control conditions in AS3809 and with a decreased expression upon 1 h of heat stress. The early heat-responsive nature of *HSFA6b* and *HSFB2b* was observed to be upregulated within 10 min of heat stress onset in barley and *HSFA6b* also showed considerable tolerance to salinity and drought stresses^23,41,42^. A decrease in expression of *TaHSFB2b* under 1.5 h of heat stress^21^ as compared to control conditions suggests that this gene performs well under non-stress conditions but its transcript level decreases after heat stress. *FaHSFC1b* gene from *Festuca arundinacea* in *Arabidopsis thaliana* plays a positive role in heat stress by upregulating heat-protective genes^43^. Upregulation of *HSFC1* genes in rice (a diploid species) by heat and oxidative stresses or a combination of these stresses^44^ corroborating with our findings from this experiment.

*HSFA3*, *HSFA5*, *HSFA9*, *HSFB2a* genes with higher expression in qRT-PCR results were also an important part of the thermotolerance profile of AS3809 though we could not find their expression in the WheatExp database. The data in the WheatExp database is from hexaploid wheat cultivar Chinese spring and TAM107 and the candidate HSFs specific to the S genome of *Ae. speltoides* could not be represented. Overexpression of homologous DREB2A from *Zea mays* in *Arabidopsis thaliana* led to the induction of HS-related genes, including *HSFA3*^45^. Downregulation of *HSFA5* in hexaploid and tetraploid wheat under heat stress has also been reported^21^. A9 of class A HSFs and subclass B3, B5 of class B HSFs is absent in monocot species like wheat and rice^20^. Contrastingly in the current investigation, *HSFA9* transcript was found in all three assemblies and is also validated by real-time PCR reaction. The results revealed the highest upregulation of this gene in AS3809 followed by PBW725 with a marginal difference. However, the role of *HSFA9* in regulating HSP expression during seed development has been demonstrated in *Arabidopsis* and sunflower^46^ though no report of seedling expression has been known. The overexpression of wheat *TaHSFB2a* in transgenic *Arabidopsis* leads to enhanced thermal and freeze tolerance^47^.

Two of the candidate HSFs, *HSFB1*, and *HSFA4b* exhibited the highest expression under heat stress in cultivated wheat, PDW274, and PBW725, respectively indicating the species-specific expression. Subclass B1 has been reported to be highly heat-inducible and its overexpression activated the expression promoter-driven reporter genes under optimal conditions^21^.

As per the protein–protein interaction network of wheat HSFs, *HSFA6b* appears to be the principal source of HSF that interacts with all other HSFs while *HSFB2a* is the ultimate target, and the rest are intermediates in the network. Thus, *HSFA6* is hypothesized to be the primary HSF/early heat-responsive transcription factor that turns on the cascade of interactions with other HSF proteins under heat stress. Our theory is supported by a prior report^21^ that demonstrated *HSFA6* members to play a key regulatory role in wheat by becoming the dominant HSFs during heat stress. It was observed that *HSFA3* interacts with *HSFA1b*. Heteromeric interactions among *HSFA1*, *HSFA2,* and *HSFA3* factors have been reported^7,48^ which enhanced target gene activation leading to acquired thermotolerance.

Phylogenetic analysis of HSF proteins of three wheat species with rice and barley revealed that the wheat HSF proteins clustered closely with respective HSFs of rice and barley^42^. All class A HSFs are grouped in one single major clade along with the C class HSFs. The class C HSF clustered with *HSFA3* indicating high similarity between these two protein classes. The class B HSFs also formed a different cluster and indicated a divergence from the type A subgroup. The clustering together of a particular class of HSFs from different species strengthens the notion of conservatory gene function of these orthologs across the species. The anomalous trend of the clustering of *HSFA3* with *HSFC1* instead of other *HSFA* clade members has been reported previously^42^.

### Differences in expression profiling of diploid, tetraploid, and hexaploid wheat

Significant differences were observed in the transcriptome expression profiling of allopolyploid wheat species, *T. durum*, and *T. aestivum* as compared to one of the wild diploid progenitors, *Ae. speltoides*. The highest expression of heat stress transcription factor genes was also observed in the diploid wild wheat, in contrast to the cultivated polyploids. Similarly, recent comparative studies^49^ focused on the expression analysis of gene pairs in the syntenic regions between hexaploid wheat chromosome 3DL and its progenitor 3L arm of *Aegilops tauschii* demonstrated 60% decreased gene expression in 70% of gene pairs in the hexaploid context of the 3DL genes. Such a reduction in gene expression has been attributed to altered interactions between transcription factors and nucleosomes on the introduction of new genomes leading to alteration in chromosome accessibility. Another study based on analyzing models of homeolog expression patterns has also demonstrated inter-genome interactions to play a key role in altered gene expression^50^. Thus, polyploidization events bring about changes in the mode of gene action that varies from additive, non-additive and dominant type in addition to the epigenetic and change in transposon activity. The expression changes observed in allopolyploids and the wild progenitor may be due to gene repression, genetic dominance, sub-functionalization, and novel activation in the former relative to the latter. The HSF genes that have been identified and validated in the current study suggest these genes play positive roles in regulating thermotolerance, especially in wild wheat. These genes are of breeding importance as their introgression into cultivars will improve their thermotolerance and thus avoiding the yield penalty due to heat stress, ultimately leading to better productivity.

## Conclusion

We generated an important genomic resource of transcriptome representing differential gene expression among three ploidies. The study highlights the conservation of gene expression across the three species as well loss of functional alleles and inter genome interactions during domestication and polyploidy as a major reason behind the reduction of expression in higher ploidies in contrast to their diploid relatives. We report the eight important HSFs in the comparative RNA-Seq analysis of three wheat species of diploid *Ae. speltoides*, tetraploid *T. durum* PDW274, and hexaploid *T. aestivum* PBW725, of which six were validated as potential novel candidates for thermotolerance from *Ae. speltoides*. Transcript profiling of HSFs under basal and heat stress conditions revealed a good consistency between the expression levels of the eight genes analyzed by qRT-PCR and their transcript levels detected using RNA-Seq in all the three wheat species. Our results encourage the exploitation of novel alleles from *Ae. speltoides* and other wild relatives for broadening the narrow genetic base of cultivated wheat.

## Materials and methods

### Plant material

Plant material included diploid wild wheat *Ae. speltoides* accession pau3809, a heat-tolerant accession, and the two cultivated wheat varieties, tetraploid *T. durum* cv. PDW274 and hexaploid *T. aestivum* cv. PBW725 which are susceptible to heat stress. Seeds of *Aegilops speltoides* accession pau3809 were originally procured from the Weizmann Institute of Science, Rehovot. PDW274 and PBW725 have been developed at PAU, India. These three germplasm lines have been maintained at PAU. Dr. Parveen Chhuneja identified *Ae. speltoides* accession pau3809 as a heat-tolerant accession and the vouchers specimen of this material has been deposited in the herbarium of National Bureau of Plant Genetic Resources (NBPGR), New Delhi, India. Experimental research and field studies on wild and cultivated varieties, including the collection of plant material, were complied with relevant institutional, national, and international guidelines and legislation. Since the plant material has been maintained by PAU, India, permissions regarding the collection of seed specimens were not required.

### RNA extraction and Illumina Sequencing

The plants of the three wheat genotypes were grown in a glasshouse maintained at night/day conditions of 18/22 °C and 80/60% relative humidity with 16 h light (500 μmol m^−2^ s^−1^) in pots containing a mixture of sand:soil: peat (3:1:1, v/v/v) at Punjab Agricultural University, Ludhiana. The 10–12 days old seedlings of the three selected wheat lines were taken during the day and immediately snap-frozen in liquid nitrogen and stored at − 80 °C for further use. Seedlings from three different plants were pooled to form one biological replicate. Total RNA from two biological replicates was extracted from the frozen seedling tissue samples using an RNA isolation kit (Qiagen) as per manufacturer protocols. The RNA concentration and purity were determined by Nanodrop™ 1000 Spectrophotometer (Thermo Scientific). Only high-quality RNA samples with OD 260/280 ranging from 1.8 to 2.2 and RIN (RNA integrity number) values ranging from 7.4 to 10.0 were used to construct the RNA-Seq library. Six separate libraries consisting of two biological replicates of the three genotypes were prepared and outsourced for transcriptome sequencing. Indexed TruSeq libraries were prepared for the six RNA samples and 100 bp paired-end sequencing was performed using a HiSeq 2000 platform. The generated raw reads were submitted to the NCBI sequence read archives (SRA) bearing accession number PRJNA767375.

### Transcriptomic data filtering and de novo assembly

**Figure 7:**
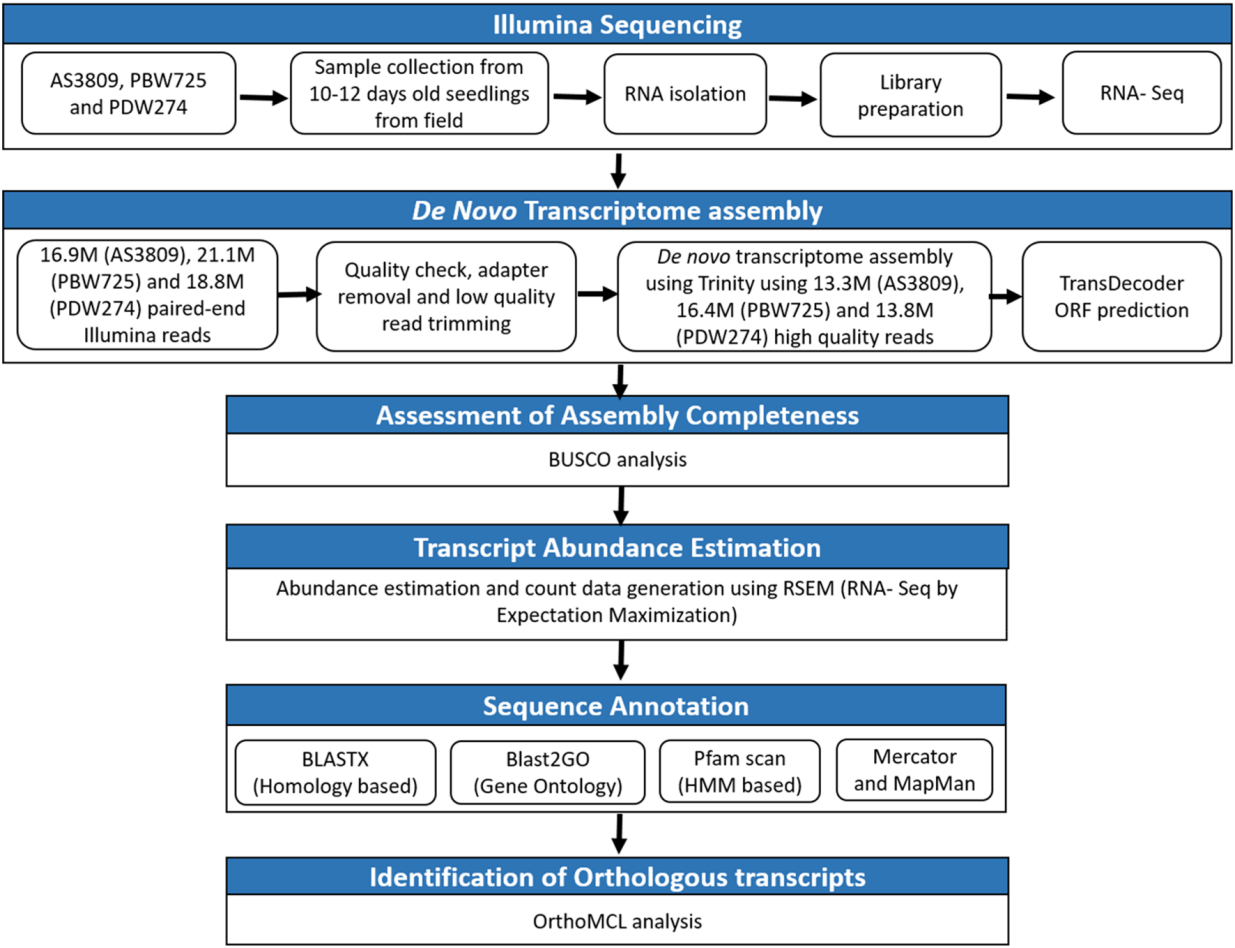
Schematic representation of de novo transcriptome reconstruction and analysis pipeline used to generate whole transcriptome assemblies for *Aegilops speltoides* acc. pau3809, *Triticum aestivum* cv. PBW725 and *Triticum durum* cv. PDW274.

The quality of reads was assessed using the FastQC toolkit v0.11.9^51^ (https://www.bioinformatics.babraham.ac.uk/projects/fastqc/). The adaptor sequences and low-quality bases (Phred score < 30) were removed from the raw reads using the Trimmomatic tool v0.38^52^ (http://www.usadellab.org/cms/?page=trimmomatic). De novo transcriptome assembly was performed for all three transcriptomes using Trinity software v2.8.4^53^ (https://github.com/trinityrnaseq/trinityrnaseq). For each of the three de novo assemblies, the transcript abundance was computed by RSEM which estimated the number of RNA-Seq fragments corresponding to each Trinity transcript, including normalized expression values as FPKM (fragments per kilobase of target transcript length per million reads mapped). The assembled transcripts from each assembly were also searched for sequence homology by performing standalone BLASTX with an e-value of 1e − 5 against publicly available NR (NCBI non-redundant protein sequences) database. A schematic representation of the de novo transcriptome reconstruction and analysis pipeline is shown in Fig. 7.

### Assessment of transcriptome assembly completeness

The comprehensive and quantitative level of completeness of transcriptome assemblies was assessed by comparing the three assembled transcript sets to a set of highly conserved single-copy orthologs using the BUSCO (Benchmarking Universal Single-Copy Orthologs) v2 pipeline^54^ (https://busco.ezlab.org/). This compared the assembled transcripts to the predefined set of 3278 Liliopsida single-copy orthologs from the OrthoDB v10 database. The number of complete, duplicated, fragmented, and missing BUSCOs were obtained in each of the three assemblies.

### Prediction of protein-coding regions and domains

TransDecoder v5.5.0 (http://transdecoder.github.io/) was used to identify all likely coding regions in the three assemblies, and the single best open reading frame (ORF) per transcript was selected as per the TransDecoder pipeline (-single best orf). The protein sequence file from TransDecoder was used to predict the protein structure domains and families of all transcripts by searching against the Protein family (Pfam) database using the HMM-based tool PfamScan^55^ (https://www.ebi.ac.uk/Tools/pfa/pfamscan/).

### Functional annotation of whole transcriptome assemblies

The identified transcripts in each of the assemblies were annotated based on corresponding homologs identified from the BLASTX program against the NCBI protein database ‘NR’ at an e-value of 1e^−5^. Based on the NR annotations, the Blast2GO v5.2.5 program (https://www.blast2go.com/) was used to obtain Gene Ontology (GO) annotations for genes in biological process, molecular function, and cellular component category for each assembly separately^56^. Mercator v3.6 (https://mapman.gabipd.org/app/mercator) was used to assign functional terms to nucleotide sequences using the MapMan ‘BIN’ ontology. MapMan was used for functional analysis and visualization^57^. The comparative GO terms for molecular function, biological process, and cellular component for each of the three assemblies were plotted using wego v2.0^58^ (https://wego.genomics.cn/).

### Identification of orthologous genes

OrthoMCL program^59^ (https://github.com/stajichlab/OrthoMCL) was used for constructing orthologous groups amongst the three wheat assemblies, using the Markov Cluster algorithm to identify (putative) orthologs and paralogs^31^. OrthoMCL was conducted for all pair-wise comparisons among the three assemblies. The output of OrthoMCL was used to determine the number of overlapping (shared across species) transcripts across the three assemblies. Further, the transcripts that were annotated/identified as heat stress transcription factors (HSFs) belonging to classes A, B, and C^60^ were retrieved from transcriptome assembly of AS3809 along with their corresponding orthologs from PBW725 and PDW274. Blast2sequence and multiple sequence alignment with ClustalX were performed to see the extent of homology between HSFs of different wheat species.

### In silico expression analysis of genes

The expression patterns of selected HSF transcripts were investigated using wheat transcriptome data from the WheatExp database (https://wheat.pw.usda.gov/WheatExp/). This database comprises RNA-Seq datasets derived from five different tissues (spike, root, leaf, grain, and stem) of hexaploid bread wheat variety Chinese Spring each sampled at three different developmental stages^61^. Another dataset consists of one-week-old seedlings of hexaploid wheat variety TAM107 treated with high temperature (40 °C) for 1 h and 6 h^62^. The 12 selected HSF transcripts were BLASTn searched with the wheat transcriptome database with an expected cut-off of 1e − 5.

### Protein–protein interaction (PPI) network analysis

The HSF protein sequences were mapped to the STRING (Search Tool for the Retrieval of Interacting Genes) database (http://string-db.org/) to acquire protein–protein interaction (PPI) networks. Active interaction sources, including text mining, experiments, databases, and co-expression as well as species limited to “*Aegilops speltoides*”. The required confidence score > 0.4 was set as the threshold to identify the PPI pairs among the HSF proteins. Cytoscape v3.6.1 s^63^ (https://cytoscape.org/) was used to visualize the PPI network.

### Phylogenetic analysis of HSF gene family

To access the phylogenetic relationships among previously identified barley and rice HSF genes^25^ with the HSF genes identified in diploid, tetraploid, and hexaploid wheat and to classify all the members of the family, multiple sequence alignment of protein sequences was done using program ClustalW. Mega 7.0 program^64^ (https://www.megasoftware.net/) was then used to construct an un-rooted neighbor-hood joining method based phylogenetic tree with 1000 bootstrap replication values with default parameters, by using multiple sequence alignment of the deduced amino acid sequence of diploid, tetraploid, hexaploid wheat along with barley and rice HSF proteins.

### Validation of HSF genes by quantitative real-time PCR (qRT-PCR)

The seeds of *Ae. speltoides* acc. pau3809, *T. aestivum* cv. PBW725 and *T. durum* cv. PDW274 were sown in germination trays in a growth chamber maintained at 22 °C/15 °C Day: night temperature, 16:8 h photoperiod. Then heat stress was given to the 10-day old seedlings at 35 °C for 1 h and immediately after stress RNA from control and heat-stressed plants was extracted for performing qRT-PCR. Primers for the 12 selected HSFs were generated using Primer Express software v3.0.1 (https://www.thermofisher.com/order/catalog/product/4363991) following the default parameters and are listed in Supplementary Table 1. Since there was considerable homology (98%) in the active coding region of the HSF genes across the three wheat species, we used the same primers for all three species. *TaActin* gene^65^ was used as the internal control. The relative gene expression levels for RT-qPCR data were calculated using the 2^−∆∆Ct^ method^66^. All reactions were conducted in triplicate.

## Supplementary Information

Supplementary Table 1.
