# Supplementary material for: Transcriptome based identification and validation of heat stress transcription factors in wheat progenitor species Aegilops speltoides
Source: Sci Rep. 2021 Nov 11;11:22049. doi: 10.1038/s41598-021-01596-6 (PMC8586331; doi:10.1038/s41598-021-01596-6)
Supplement: Supplementary file 1 — Supplementary Table 1. [file 41598_2021_1596_MOESM1_ESM.docx]

**Transcriptome based identification and validation of heat stress transcription factors in wheat progenitor species *Aegilops speltoides***

**Sushmita Seni^1^, Satinder Kaur^1^*, Palvi Malik^1^, Inderjit Singh Yadav^1^, Parul Sirohi^2^, Harsh Chauhan^2^, Amandeep Kaur^1^ and Parveen Chhuneja^1^**

**Affiliations**

^1^School of Agricultural Biotechnology, Punjab Agricultural University, Ludhiana, Punjab, India-141004

^2^Department of Biosciences and Bioengineering, Indian Institute of Technology Roorkee, Roorkee, Uttarakhand, India-247667

**ORCID ID:**

SK: 0000-0003-3704-3074

PM: 0000-0003-1215-7120

AK: 0000-0002-9140-3424

PC: 0000-0002-8599-9479

*Corresponding author email: [satinder.biotech@pau.edu](mailto:satinder.biotech@pau.edu)

**Supplementary Table 1: Sequence of primers designed for Heat Stress Transcription Factors (HSFs) for real-time PCR amplification**

| **Primer ID** | **Forward Primer (5ˈ–3ˈ)** | **Reverse Primer(5ˈ–3ˈ)** |
| --- | --- | --- |
| HSFA1B | GATGGTTTCCTTCTGTTGATGGT | GCTCAACACC.TACGGATTCAG |
| HSFA2 | CGCCGTTCCTGACGAAGA | CGCTCCACGACATGATGCT |
| HSFA3 | CCCCAATGTCCTCTGATGCT | CATGCTGCCGGGTAGCTT |
| HSFA4B | CACC.AGGAGGCCCAATCA | CGGTCTCCTGCTGTTGTTTGA |
| HSFA5 | CCTTCATGCCCTGGAGGAT | CCCAAAGCTCGCACGAA |
| HSFA6B | TTGCCAAGAACC.CCATCATC | TCGTAGCCTGCCTTCCATG |
| HSFA9 | AGCTTCATACGCCAGCTCAAC | GGCCCACTCCCATCTATCAG |
| HSFB1 | GCTCGGCGGACC.AAGAG | GACGCGTCCTTGAGGATCAC |
| HSFB2A | AGCTCGGTCAGATGAAGAAGCT | CGTCGGTGTACTTGGACATGA |
| HSFB2B | GCGTCTCCATAGGGCTCAAG | TCCTCCCTGTCGTCGTCTTC |
| HSFB3 | TGGCATGCACGTAAGGATGT | GTACGGCCGAGACAGCAAA |
| HSFC1B | TCCCCTCGGCATAGTTGCTA | CGTGGCTGGAGTGACTACTATGG |
